# A Cohort Study of Antihyperglycemic Medication Exposure and Gastric Cancer Risk

**DOI:** 10.3390/jcm9020435

**Published:** 2020-02-05

**Authors:** Audrius Dulskas, Ausvydas Patasius, Auguste Kaceniene, Donata Linkeviciute-Ulinskiene, Lina Zabuliene, Giedre Smailyte

**Affiliations:** 1 Department of Abdominal and General Surgery and Oncology, National Cancer Institute, 1 Santariskiu Str., LT-08406 Vilnius, Lithuania; 2 University of Applied Sciences, Faculty of Health Care, 45 Didlaukio Str., LT-08303 Vilnius, Lithuania; 3 Institute of Clinical Medicine, Faculty of Medicine, Vilnius University, 21/27 M. K. Ciurlionio Str., LT-03101 Vilnius, Lithuania; linazabuliene@gmail.com; 4 Laboratory of Cancer Epidemiology, National Cancer Institute, 1 Santariskiu Str., LT-08406 Vilnius, Lithuania; ausvydas.patasius@nvi.lt (A.P.); auguste.kaceniene@nvi.lt (A.K.); giedre.smailyte@nvi.lt (G.S.); 5 Institute of Health Sciences, Faculty of Medicine, Vilnius University, 21/27 M. K. Ciurlionio Str., LT-03101 Vilnius, Lithuania; 6 Institute of Biomedical Sciences, Faculty of Medicine, Vilnius University, 21/27 M. K. Ciurlionio Str., LT-03101 Vilnius, Lithuania; linkeviciutei@gmail.com

**Keywords:** gastric cancer, metformin, diabetes, population-based study, antihyperglycemic therapy, insulin, sulfonylurea

## Abstract

We assessed gastric cancer risk in type 2 diabetes mellitus patients. Gastric cancer patients with diabetes between 2001–2012 were identified. Four groups were analysed: combination therapy with metformin users; insulin and other medication users; metformin and insulin users; and sulfonylurea users. Standardised incidence ratios (SIRs) for gastric cancers as a ratio of the observed number of cancer cases in people with diabetes to the expected number of cancer cases in the underlying general population were calculated. A total of 99,992 patients with diabetes were analysed and 337 gastric cancer cases in patients with diabetes were observed when compared to the expected number of 400.54 gastric cancer cases, according to the cancer rates of the general population (SIR 0.84, 95% confidence interval (CI): 0.76–0.94). Lower risk of gastric cancer was found both in male and female patients with diabetes, however, risk among females was insignificantly lower. Higher gastric cancer risk was found in the group of diabetic patients treated with sulfonylureas (SIR 1.31, 95% CI: 1.04–1.65) and significantly lower risk than expected from the general population was found in the group of metformin users (SIR 0.75, 95% CI: 0.66–0.86). Type 2 diabetes mellitus was not associated with increased risk of gastric cancer. Metformin might decrease the risk of gastric cancer in patients with diabetes, while sulfonylureas may increase gastric cancer risk.

## 1. Introduction

Gastric cancer is a deadly disease with over 1,000,000 new cases in 2018 and an estimated 783,000 deaths (equating to one in every 12 deaths globally), making it the fifth most frequently diagnosed cancer and the third leading cause of cancer death [1]. Many environmental and genetic risk factors are known in the development of gastric cancer including *Helicobacter pylori (H. pylori)* infection, dietary habits and lifestyle with increased salting of food, low fresh fruit and vegetable intake, alcohol consumption and smoking, obesity, Barret’s oesophagitis, gastric diseases, and other [2]. 

The International Diabetes Federation (IDF) estimated that one in 11 adults aged 20–79 years (463 million adults) had diabetes mellitus globally in 2018 [3]. Obesity and type 2 diabetes mellitus have been linked to many types of cancer including gastric. This association has been primarily attributed to insulin resistance and cluster factors of metabolic syndrome, which also play an additive carcinogenic role [4].

Type 2 diabetes mellitus can be treated with monotherapy or various therapeutic combinations including metformin, sulfonylureas, thiazolidinediones, dipeptidyl peptidase-4 inhibitors (DDP-4), glucagon like peptide-1 (GLP-1) agonists, sodium-glucose co-transporter-2 (SGLT-2) inhibitors, or insulin. Metformin is the first-line treatment for type 2 diabetes [5]. Metformin has the ability to counteract insulin resistance, reduce fasting hyperglycaemia without weight gain or increased risk of hypoglycaemia [6]. Metformin acts on several mechanisms associated with cancer development and progression, and may directly inhibit tumour growth [7]. Some authors have hypothesised that the benefits of metformin on cancer among patients with type 2 diabetes mellitus are mediated by upregulation of MiR26b induced by metformin via reduction of insulin resistance [6,8,9]. In vitro models suggest that the underlying mechanism of metformin is the suppression of the mammalian target of rapamycin (mTOR) by activation of the liver kinase B1 (LKB1) dependent adenosine 5-monophosphate-activated protein kinase (AMPK) pathway [10]. Upregulation of AMPK directly suppresses mTOR, resulting in the inhibition of protein synthesis in cancer cells and cell proliferation [11].

Previously, we found that diabetic status had no effect on colorectal cancer specific and overall survival and, furthermore, antihyperglycemic treatment with metformin might have a positive effect on colorectal cancer by decreasing the incidence risk and prolonging the overall and cancer specific survival [12,13]. In another study, we showed that metformin might also have a positive effect on gastric cancer survival compared to other medication groups [14].

Previous findings have suggested that, among antihyperglycemic medications, insulin and sulfonylureas may increase the risk of cancer by interacting with insulin and insulin growth factor-1 (IGF-1) receptor signalling, which enhances proliferation and carcinogenesis [15,16]. Insulin itself is a growth factor and has metabolic and mitogenic effects [17,18]. Hyperinsulinemia, especially in the presence of insulin resistance, may promote cancer cell growth either through the insulin receptor or IGF-1 receptor. Cancer cell growth may also be engaged via increased bioavailability of free IGF-1 by inhibition of IGF binding proteins. Some reviews have shown that sulfonylureas might increase the level of insulin, which has direct and indirect actions on tumours. Sulfonylureas might also be associated with a greater risk of cancer compared to DPP-4 inhibitors and TZDs [19]. On the other hand, according to a recent review, some sulfonylureas have a protective effect against cancer by reducing the growth of cancer cells, inhibiting tumour necrosis factor (TNF) production by human peripheral blood mononuclear cells and by inhibition of TNF bioactivity and immunoreactivity in mice serum [20].

So far, only slightly more than 10 studies, most of them coming from Asian countries, analysing gastric cancer and antihyperglycemic medication (mainly metformin) associations have been published [21,22,23,24,25,26,27,28,29,30,31]. Furthermore, these results are still contradictory: from studies showing no effect on gastric cancer [21,22,23,24,25] to decreased risk for gastric cancer [26,27,28,29,30,31]. 

The main objective of our study was to evaluate gastric cancer risk in type 2 diabetes mellitus patients and the effect of antihyperglycemic therapy on gastric cancer risk.

## 2. Research Design and Methods

### 2.1. Data Sources and Study Design

All procedures followed were in accordance with the ethical standards of the responsible committee on human experimentation (institutional and national) and with the Helsinki Declaration of 1964 and later versions. The study was approved by the National Cancer Institute Review Board.

A retrospective cohort design was used to examine the relationship between diabetes and gastric cancer risk. All data used in this study comprised individual cancer patient records and were provided by the Lithuanian Cancer Registry and the National Health Insurance Fund (NHIF). The cohort was composed of patients identified from the National Health Insurance Fund (NHIF) database, with diagnosis of type 2 diabetes mellitus. The NHIF collects demographic data and entries on the primary and secondary healthcare services provided, emergency and hospital admissions, and prescriptions of reimbursed medications. Information regarding the diagnosis of type 2 diabetes (International Classification of Diseases (ICD)-10 code E11) and diabetes treatment were obtained from the NHIF. Patients with a diagnosis of type 2 diabetes mellitus during 2001–2012 were identified for the study. As in the database, diagnoses of admission were registered manually; to increase the specificity of type 2 diabetes mellitus cases, only patients who had more than six prescriptions for reimbursed antihyperglycemic medications were included in the study. 

Cancer cases were identified by record linkage with the Lithuanian Cancer Registry, which is a nationwide population-based cancer registry that contains personal and demographic data and information on the type of all diagnosed cancer cases in Lithuania since 1978. 

We analysed only type 2 diabetes cases, diagnosed from the age of 40. All patients with gastric cancer and any other cancer type prior to type 2 diabetes mellitus diagnosis were excluded from the cohort and finally 99,992 patients with type 2 diabetes mellitus were analysed. Only primary gastric cancers (according to ICD-10, codes C16) detected more than six months after type 2 diabetes mellitus diagnosis were considered in the risk analysis. 

### 2.2. Exposure Definition

Cohort members were classified into four groups according to treatment: “metformin and other” medication (except insulin) users; “insulin and other” medication (except metformin) users; “metformin and insulin” users; and “sulfonylurea” users. Other medications included all other oral antihyperglycemic agents. The study cohort was restricted to patients who had used antihyperglycemic medications. This latter restriction was necessary to ensure that all patients actually had type 2 diabetes mellitus. Patients not reported with a diabetes diagnosis in the NHIF database were classified as non-diabetic. Exposure to antihyperglycemic medications was identified from linked prescription data. Exposure (yes/no) was defined according to whether or not the individual had a supply of antihyperglycemic medications available at any point. Six months were defined as the shortest duration of exposure required for effect.

### 2.3. Statistical Methods

The person–time of observation was computed from the date of the first recorded type 2 diabetes mellitus diagnosis in the NHIF database until diagnosis of gastric cancer, emigration, death or end of the observation period (31 December 2012), whichever came first. 

We calculated standardised incidence ratios (SIRs) for gastric cancers as a ratio of observed number of cancer cases in people with type 2 diabetes mellitus diagnosis to the expected number of cancer cases in the underlying general population. Expected numbers were calculated as the multiplication of the exact person–years under observation in the cohort by sex-, calendar year- and 5-year-age-group-specific national gastric cancer incidence rates. The 95% confidence intervals (CI) for the SIRs were estimated, assuming that the number of observed cases follows a Poisson distribution. 

We computed the SIRs by sex, age of diabetes diagnosis, and use of antihyperglycemic medications. All statistical analyses were carried out using STATA 11 statistical software (StataCorp. 2009. Stata Statistical Software: Release 11.0. College Station, TX, USA).

## 3. Results

Overall, we analysed 99,992 patients with type 2 diabetes mellitus in Lithuania between 2001 and 2012. A total of 39,255 males and 60,737 females were included in the final cohort. Table 1 presents the observed number of gastric cancer cases, the SIRs of developing gastric cancer together with 95% confidence intervals by age, and by antihyperglycemic medication use. Overall, 337 gastric cancer cases in patients with diabetes were observed, compared to the expected number of 400.54 gastric cancer cases, according to the cancer rates of the general population (SIR 0.84, 95% confidence interval (CI): 0.76–0.94). Lower risk of gastric cancer was found in both male and female patients with type 2 diabetes mellitus, however, cancer risk among females was insignificantly lower. There were no significant differences in risk of gastric cancer by age at type 2 diabetes mellitus diagnosis, except for the 60–69 years old female patient group, where gastric cancer risk was significantly lower than expected from the general population (SIR 0.77, 95% CI: 0.64–0.93). 

Higher gastric cancer risk was found in the group of diabetic patients treated with sulfonylureas (SIR 1.31, 95% CI: 1.04–1.65) as well as insulin and other medications (SIR 1.16, 95% CI: 0.73–1.84), although the latter result was insignificant. Significantly lower risk than expected from the general population was found in the group of metformin users (SIR 0.75, 95% CI: 0.66–0.86) and insignificantly lower risk was found for metformin and insulin users (SIR 0.67, 95% CI: 0.40–1.11). 

## 4. Discussion

Our study revealed that type 2 diabetes mellitus was not associated with increased risk of gastric cancer (SIR 0.84, 95% CI: 0.76–0.94). We also found that metformin might decrease the risk of gastric cancer in patients with type 2 diabetes mellitus, since significantly lower risk than expected from the general population was found in the group of metformin users (SIR 0.75, 95% CI: 0.66–0.86). In contrast, an epidemiologic study from Taiwan by Lee et al. who assessed 800,000 patients with multiple site cancers including gastric cancer showed that metformin use might increase gastric cancer risk (Hazard ratio (HR), 1.41; 95% CI: 0.42–4.73) [21]. Another study also from Taiwan by Hsieh et al. compared 61,777 patients with type 2 diabetes mellitus and 677,378 subjects without diabetes and found that patients with type 2 diabetes mellitus had insignificantly lower risk of gastric cancer (patients with diabetes vs. without diabetes odds ratios adjusted by age and sex (OR), 0.920; 95% CI: 0.836–1.012, *p* = 0.088) [22]. Neither metformin nor sulfonylureas had any effect on gastric cancer risk (insulin vs. metformin (OR), 1.855; 95% CI: 0.779–4.419 and sulfonylurea vs. metformin (OR), 1.547; 95% CI: 0.923–2.594) [22]. Chen et al. found that during the first four years after diabetes diagnosis, the incidence of gastric cancer was relatively low in diabetic patients (HR 0.63; 95% CI: 0.42–0.97). However, after that time, the diabetic group had a 76% (95% CI: 1.06–2.91) higher risk of developing gastric cancer than the comparison group [23]. Like us, other researchers have assessed the effect of different antihyperglycemic medications (insulin, metformin, sulfonylureas, thiazolidinediones, alpha-glucosidase inhibitors, non-sulfonylurea insulin secretory analogues) on gastric cancer risk [23]. The study found that all medications except alpha-glucosidase inhibitors had no effect on the gastric cancer risk, while alpha-glucosidase inhibitors were associated with a significantly decreased risk of gastric cancer (adjusted HR 0.38; 95% CI: 0.15–0.96) [23]. Another nationwide population-based study from Taiwan found that patients with diabetes mellitus had an increased risk of gastric cancer (HR 1.49; 95% CI: 1.16–1.92, *p* = 0,002) and may be affected by the use of different categories of glucose-lowering therapies: insignificant effect on gastric cancer risk in diabetes treatment with sulfonylurea use (HR 1.13; 95% CI: 0.68–1.86), with metformin use (HR – 1.28; 95% CI: 0.72–2.08), or with insulin use (HR 0.53; 95% CI: 0.27–1.04), but gastric cancer risk significantly decreased with thiazolidinedione use (HR 0.11; 95% CI: 0.02–0.82) [24]. 

Unlike our results, which showed that the group of diabetic patients treated with sulfonylureas had a higher than expected gastric cancer risk (SIR 1.31, 95% CI: 1.04–1.65), Chang et al. found that sulfonylurea had no effect on gastric cancer risk (crude OR 0.85; 95% CI: 0.64–1.12), meanwhile insulin treatment increased the risk of gastric cancer (crude OR 2.43 95% CI: 1.89–3.13) [25]. 

In comparison, there are studies showing a lowering of gastric cancer risk with use of some of the antihyperglycemic medications [26,27,28,29,30,31]. Valent et al., similarly to us, found that the total number of metformin prescriptions was associated with significant, but minor, reduced risk of most types of digestive cancers (gastric cancer HR 0.99; 95% CI: 0.986–0.994) [26]. However, unlike our results, sulfonylurea use was associated with a reduced risk of gastric cancer (HR 0.989; 95% CI: 0.981–0.997) [26]. Interestingly, a recent Korean study by Kim et al. showed that the relative risk of gastric cancer development among metformin users was reduced by even up to 43% (HR 0.57; 95% CI: 0.37–0.87) after three years or more of metformin use [28]. Two recent meta-analyses and systemic reviews have shown conflicting results [32,33]. A meta-analysis by Miao et al. of 22 studies showed that people with diabetes mellitus had little or no change in the risk of gastric cancer or gastric cancer mortality [32]. Another meta-analysis by Zhou et al., which included seven studies, found that metformin therapy was associated with a reduction in the risk of gastric cancer in patients with type 2 diabetes mellitus [33].

Our study has several advantages: it has a large sample size, which reduces the likelihood of random error; includes the entire regional population, thus minimizing selection bias; and it does not rely on self-reports.

Obviously, our cohort study has some limitations. First, there was a relatively small number of patients with gastric cancer and type 2 diabetes mellitus. Second, we could not adjust for confounding factors like body mass index, smoking history, lifestyle, dietary habits, and *H. pylori* infection as this data were unavailable. 

## 5. Conclusions

To conclude, type 2 diabetes mellitus was not associated with increased risk of gastric cancer. Metformin might decrease the risk of gastric cancer in patients with type 2 diabetes mellitus, while sulfonylureas might be associated with increased gastric cancer risk.

## Figures and Tables

**Table 1:** Numbers of observed (Obs) and expected (Exp) cases of gastric cancers, standardised incidence ratios (SIR) with 95 percent confidence intervals (CI) in type 2 diabetes patients.

	Male					Female						Overall				
	Obs	Exp	SIR	95% CI		Obs	Exp	SIR	95% CI		Obs		Exp	SIR	95% CI	
Overall	177	216.80	0.82	0.70	0.95	160	183.74	0.87	0.75	1.02	337		400.54	0.84	0.76	0.94
Age at diagnosis																
40–50	15	12.08	1.24	0.75	2.06	4	5.50	0.73	0.27	1.94	19		17.58	1.08	0.69	1.69
50–59	40	50.63	0.79	0.58	1.08	21	26.73	0.79	0.51	1.21	61		77.35	0.79	0.61	1.01
60–69	66	82.18	0.80	0.63	1.02	43	59.58	0.72	0.54	0.97	109		141.77	0.77	0.64	0.93
≥70	56	71.91	0.78	0.60	1.01	92	91.93	1.00	0.82	1.23	148		163.83	0.90	0.77	1.06
Antihyperglycemic medication																
Metformin and other	120	163.23	0.74	0.61	0.88	110	142.88	0.77	0.64	0.93	230		306.11	0.75	0.66	0.86
Insulin and other	10	10.27	0.97	0.52	1.81	8	5.22	1.53	0.77	3.06	18		15.49	1.16	0.73	1.84
Metformin and insulin	11	13.03	0.84	0.47	1.52	4	9.42	0.42	0.16	1.13	15		22.45	0.67	0.40	1.11
Sulfonylureas	36	30.27	1.19	0.86	1.65	38	26.21	1.45	1.05	1.99	74		56.49	1.31	1.04	1.65

Obs—observed number of patients; Exp—expected number of patients; SIR—standardised incidence ratio; CI—confidence interval.
